# Maternal metabolomic profiling and congenital heart disease risk in offspring: A systematic review of observational studies

**DOI:** 10.1002/pd.6301

**Published:** 2023-01-26

**Authors:** Stuart Mires, Snigdha Reddy, Clare Skerritt, Massimo Caputo, Kelly‐Ann Eastwood

**Affiliations:** ^1^ University of Bristol Bristol UK; ^2^ University Hospitals Bristol and Weston NHS Foundation Trust Bristol UK; ^3^ Birmingham Women's and Children's NHS Foundation Trust Birmingham UK

## Abstract

Aetiological understanding and screening methods for congenital heart disease (CHD) are limited. Maternal metabolomic assessment offers the potential to identify risk factors and biomarkers. We performed a systematic review (PROSPERO CRD42022308452) investigating the association between fetal/childhood CHD and endogenous maternal metabolites. Ovid‐MEDLINE, Ovid‐EMBASE and Cochrane Library were searched between inception and 06/09/2022. Case control studies included analysing maternal blood or urine metabolites in pregnancy or postpartum where there was foetal/childhood CHD. Risk of bias assessment utilised the Scottish Intercollegiate Guidelines Network methodology checklist and narrative synthesis was performed. A total of 134 records were screened with eight eligible studies (*n* = 3242 pregnancies, *n* = 842 CHD‐affected offspring). Five studies performed metabolomic analysis in pregnancy. Metabolites distinguishing case and control groups spanned lipid, glucose and amino‐acid pathways, with the development of sensitive risk prediction models. No single metabolite consistently distinguished cases and controls across studies. Three studies performed targeted analysis postnatally with altered lipid and amino acid metabolites and raised homocysteine and markers of oxidative stress identified in cases. Included studies reported small sample sizes, analysing different biosamples at variable time points using differing techniques. At present, there is not enough evidence to confidently associate maternal metabolomic profiles with offspring CHD risk. However, several identified pathways warrant further investigation.

## Key points

**What's already known about this topic?**

- Congenital heart disease (CHD) is the most common congenital anomaly.
- Antenatal detection rates remain low, with limited understanding of aetiology.
- Metabolomics provides a global physiological overview to derive potential novel biomarkers in screening and causation.

**What does this review add?**

- Several metabolites in lipid, amino acid and homocysteine/methionine metabolic pathways significantly differ in mothers with offspring affected by CHD.
- We identify metabolic pathways for further investigation in establishing causality and screening biomarkers for foetal CHD.

## 1 INTRODUCTION

Globally, congenital anomalies affect over 2% of total births, with congenital heart disease (CHD) the most common. CHD prevalence is increasing with similar rates reported across Europe.^1^, ^2^ In England, CHD represents one third of congenital anomalies, with severe CHD, generally defined as requiring surgery or treatment in the first year of life, accounting for around half of these.^3^ Increasing detection, survival and the need to reduce postoperative mortality in infants with severe and critical CHD emphasise the importance of adequate understanding of CHD pathology on a global level.^4^

Cardiac development begins in the second human embryonic week, with the critical period recognised as weeks two to eight.^5^, ^6^ Our understanding of CHD aetiology is significantly limited. Around 15% of CHD can be linked to a known genetic cause including aneuploidy, such as trisomy 21 (8%–10%) and single gene defects such as Noonan's syndrome (3%–5%). Approximately 2% of cases are associated with specific environmental factors including maternal medical conditions, for example, diabetes mellitus or phenylketonuria.^7^ A meta‐analysis and umbrella review of literature has also linked numerous other maternal and environmental factors to foetal CHD including obesity, smoking, infection, air pollution and recreational and prescription drug use.^8^, ^9^ Despite such associations, mechanistic understanding is lacking with most CHD causative factors remaining unexplained.

Prenatal screening evaluates individual risk in obstetrics. This is demonstrated by aneuploidy screening, combining foetal nuchal translucency (NT) measurement on ultrasound with maternal serum biomarkers and age.^10^ Combined screening tests generally provide more accurate assessment of risk than individual markers alone. Diagnostic genetic testing by invasive sampling of the placenta or amniotic fluid provides an accurate result but carries a risk of miscarriage of approximately 0.5%.^11^ More recently, non‐invasive prenatal testing where analysis of maternal serum is undertaken for cell‐free foetal DNA has revolutionised prenatal screening for aneuploidy with a significantly higher sensitivity and specificity and no associated risk of miscarriage.^12^, ^13^ Whilst these technological developments are being extended to single gene disorders, they are unlikely to provide adequate screening for CHD given its multifactorial aetiology and low single gene causality yield.

The focus of prenatal screening for congenital anomalies primarily rests on ultrasonographic assessment. Increased NT, abnormal ductus venosus flow and tricuspid regurgitation in the first trimester are associated with an increased risk of CHD.^14^ The foetal anomaly screening programme recommends a systematic anatomy scan between 18 and 21 weeks, aiming to identify common and major congenital anomalies including serious cardiac anomalies (transposition of the great arteries, atrioventricular septal defects, tetralogy of Fallot and hypoplastic left heart syndrome).^10^ Patient uptake is over 98%, illustrating the importance of this screening to pregnant woman.^15^ However, targeted detection rates for CHD are only 50%.^10^ Recent data in England shows that this detection rate is accurate, with 54.5% of major CHD diagnosed antenatally.^3^ Ultrasound has several limitations including intraoperator variability, foetal factors such as position or multiple pregnancy and maternal factors such as obesity. Foetal echocardiography, provided by trained foetal cardiologists, provides greater sensitivity for CHD detection but is generally only performed when an anomaly is suspected on screening ultrasound or in screening high‐risk populations.^16^, ^17^ Foetal echocardiography can improve detection of CHD following a normal complete foetal anomaly scan; however, performance of 750 scans is required to detect one additional case.^18^ Foetal echocardiography is costly and requires significant expertise.^19^ As such, there are significant limitations of current antenatal screening strategies for foetal CHD.

Metabolomic analysis utilising chromatography and mass spectrometry or nuclear magnetic resonance spectroscopy detects compounds such as carbohydrates, amino acids, lipids and vitamins in biosamples such as blood or urine.^20^ Such profiles reflect global physiology, influenced by genetics, the environment and physiological stressors.^20^, ^21^, ^22^ Use of machine learning techniques in large biological datasets has revolutionised utilisation of such information.^23^ In CHD, it is hypothesised that changes in foetal perfusion or organ function could be reflected in maternal metabolic alterations. As such, metabolomic analyses provide the potential to derive novel biomarkers to screen for CHD.^21^ However, the presence of foetal cardiovascular shunts and the placental interface may limit this. Furthermore, elucidating potential maternal metabolic risk factors could help improve mechanistic understanding and screening for CHD.

A systematic review assessing altered endogenous maternal metabolite levels in maternal screening or risk factor profiling for foetal CHD has not been undertaken previously. We therefore aim to critically appraise available literature to assess the association between altered endogenous maternal metabolite levels and foetal or childhood CHD. Our findings will inform subsequent recommendations for future research and practice.

## 2 METHODS

### 2.1 Protocol, search strategy and eligibility criteria

The systematic review protocol was registered a priori at PROSPERO (registration number CRD42022308452) and followed the PRISMA 2020 checklist.^24^ A systematic literature search of electronic databases MEDLINE (via OVID), EMBASE (via OVID) and the Cochrane library was conducted to identify relevant studies published between the inception of each database and February 3rd 2022 without language restrictions. Searches were re‐run prior to final submission (6th September 2022). Reference lists of included studies were manually screened and experts contacted to identify additional emerging and relevant literature. Figure S1 outlines the search strategy for each database. Observational studies were included. It was not anticipated that randomised control or control trials would be available. Case reports and case series were excluded due to high risk of bias.

Case inclusion criteria were mothers of fetuses/children with CHD. Studies with surrogate mothers or pregnancies with egg donation were excluded due to the potential influence of the surrogate or donor environment on the metabolome. The exposure was maternal blood or urine metabolite analysis in pregnancy or postpartum. The primary outcome was foetal/childhood CHD.

### 2.2 Study selection, data extraction and quality assessment

SM and SR independently screened titles and abstracts to exclude studies that did not meet inclusion criteria. Agreement on potential inconsistencies was reached by consensus. Data extraction from selected studies was performed by SM and checked by SR. Risk of bias assessment was assessed by SM and SR independently utilising the Scottish Intercollegiate Guidelines Network case control study checklist and guidance notes.^25^ This facilitates assessment of subject selection, exposure assessment, confounding and statistical analysis. Studies are assessed overall as high quality (++), acceptable (+) or unacceptable. Unacceptable studies were excluded from analysis due to high risk of bias.

### 2.3 Data synthesis and analysis

A narrative synthesis was performed including tabulation of study characteristics and results. Quantitative synthesis of data and meta‐analysis was precluded due to variation in studies including biomaterial analyses, timing of sampling and metabolic analyses performed. Metabolites distinguishing case and control populations identified through machine learning and confounder adjusted analyses were summarised. For antenatal studies reporting risk prediction, the models developed and included biomarkers are presented.

## 3 RESULTS

### 3.1 Literature search

**FIGURE 1:**
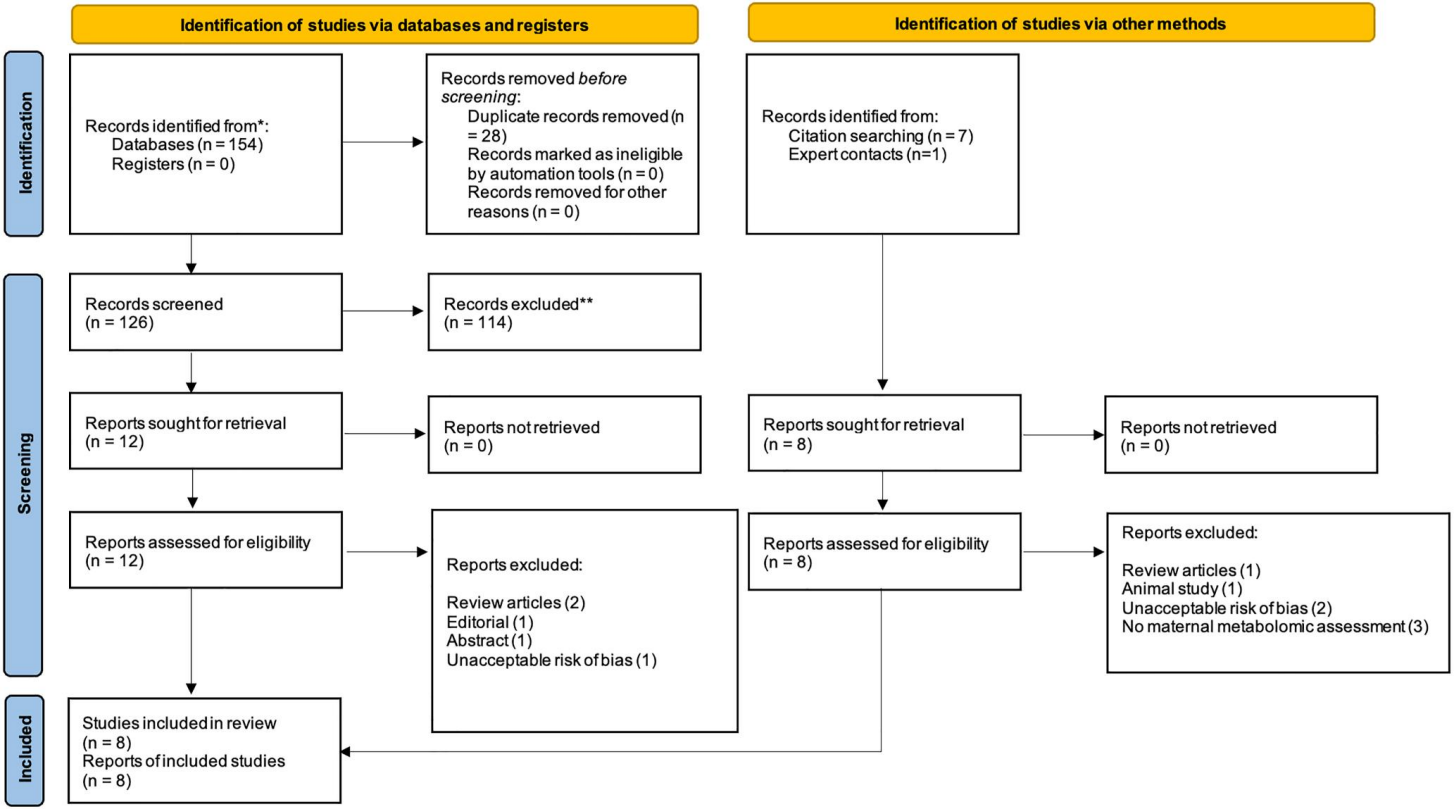
PRISMA 2020 flow diagram of study identification, screening, inclusion and exclusion.^57^

Figure 1 demonstrates a PRISMA flow diagram of included studies. Following systematic literature searching, 134 unique studies were identified. 114 studies were excluded after title and abstract screening. Full text reports were reviewed for remaining studies. A further 12 were excluded, with reasons outlined in Figure 1. This resulted in 8 studies included.^26^, ^27^, ^28^, ^29^, ^30^, ^31^, ^32^, ^33^

### 3.2 Characteristics of included studies

Table 1 summarises the characteristics and risk of bias assessment for the 8 included studies. Primary metabolomic analysis data from 3242 pregnancies is included, with 842 having a child affected by CHD. All studies were case control design published between 2005 and 2022. Studies originated from 4 countries: USA,^4^ UK,^2^ Italy^1^ and China.^1^

**TABLE 1:** Summary study characteristics from included studies.

Study design	Maternal/fetal characteristics	Analysis	Outcome	Risk of bias/methodology checklist^a^
Bahado‐Singh et al. 2014^26^ Country UK Case control *n* = 27 cases *n* = 59 controls Source Blood (serum) Sample Timing Antenatal: 11‐13 weeks’ gestation	Case inclusion Isolated major cardiac defect (lethal or requires surgery/catheter 1^st^ year life) Control inclusion No pregnancy complications Paired (same day sampling to a control) Case/control exclusion Aneuploidy Non‐cardiac anomaly	Analysis DI/LC‐MS‐MS (targeted) and NMR metabolomics. Number metabolites identified 150 (DI/LC‐MS‐MS) 38 (NMR) 174 distinct metabolites	CHD diagnosis method Fetal echocardiography. Postnatal physical examination. Diagnosis timing Antenatal CHD diagnoses (n) AVSD/DORV (1) AVSD/DORV/PA (1) DORV/PS (2) DORV/ToF (2) DORV/PA (1) TGA (3) TGA‐corrected VSD (1) TGA/PS (1) ToF (9) ToF/MS (1) ToF/PA (5) 100% EUROCAT Severe CHD	+
Friedman et al. 2021^27^ Country USA Case control *n* = 36 cases *n* = 41 controls Source Urine Sample timing Antenatal: Case median 157 days Control median 151 days	Case inclusion Isolated CHD 18‐50 years old. Control inclusion No anomaly suspected Gestational age within 2 weeks case 18‐50 years old. Paired (same day sampling to a control) Case/control exclusion Aneuploidy Non‐cardiac anomaly Multifetal gestation Lack of capacity	Analysis H NMR and LC‐MS‐MS (targeted) metabolomics. Number metabolites identified 87 (LC‐MS‐MS) 135 (NMR) 206 distinct metabolites	CHD diagnosis method Not stipulated Diagnosis timing Antenatal or postnatal CHD diagnoses (n) Aortic coarctation (1) Aortic root dilation (1) APVM (1) AS (1) ASD (3) AV hypoplasia (1) AVSD (1) DORV (2) Hypoplastic aorta (1) HLHS (6) HRHS (2) Mitral atresia (1) Overriding aorta (1) PA (3) PS (1) RAA with vascular ring (2) Single ventricle (1) TA (2) TGA (1) ToF (3) Truncus arteriosus (1) VSD (18) 27.8% EUROCAT Severe CHD	+
Troisi et al. 2021^28^ Country Italy Case control *n* = 70 cases *n* = 280 controls Source Blood (serum) Sample timing Antenatal Case median 147 days Control median 140 days	Case inclusion Voluntary TOP for cardiac anomaly. Control inclusion No anomaly postnatal examination. Case/control exclusion Other malformations. Infections – syphilis, hepatitis B, rubella, CMV, toxoplasmosis, herpes simplex. Multifetal gestation In Vitro fertilisation Chronic maternal conditions – diabetes, hypertension, renal disease.	Analysis Untargeted GC‐MS metabolomics. Number metabolites identified Not specified	CHD diagnosis method Fetal ultrasound with post mortem confirmation. Diagnosis timing Antenatal CHD diagnoses (n) Aortic coarctation (3) ASD (6) AVSD (2) Complex CHD (40) Di George syndrome (2) Single ventricle (6) ToF (7) VSD (4) 82.9% EUROCAT Severe CHD (assuming complex is severe)	+
Xie et al. 2019^29^ Country China Case control *n* = 70 cases *n* = 70 controls Source Urine Sample timing Antenatal Case mean 28.27 weeks Control mean 30.76 weeks	Case inclusion 4D ultrasound diagnosis CHD. 22‐24 or 30‐32 weeks. Control inclusion No CHD, confirmed after birth. Case/control exclusion Antibiotic use in the past 3 months. TOP. Maternal conditions – diabetes, liver or renal disease, thyroid disease. Aneuploidy. Family history. Picky eating.	Analysis Untargeted GC‐MS metabolomics. Number metabolites identified 220 (GC‐MS)	CHD diagnosis method 4D fetal echocardiography Diagnosis timing Antenatal CHD diagnoses (n) Aortic abnormalities (8) AVSD (5) Complex CHD (5) DORV (2) Endocardial cushion defect (3) Pulmonary arterial anomaly (3) Single atrium/ventricle (4) ToF (10) TR (3) VSD (27) 52.9% EUROCAT Severe CHD (assuming complex and aortic is severe)	+
Taylor et al. 2022^33^ Country UK Case control Mass spectrometry: *n* = 46 cases *n* = 2559 controls NMR (validation): *n* = 87 cases *n* = 7296 controls Source Blood (plasma) Sample timing Antenatal dataset 1: Case mean 26.2 weeks Control mean 25.8 weeks Antenatal dataset 2: Case mean 26.2 weeks Control mean 26.0 weeks	Case inclusion CHD. Control inclusion No anomaly suspected. Case/control exclusion Multiple pregnancy.	Analysis Untargeted UPLC‐MS/MS, HILIC/UPLC‐MS/MS metabolomics. NMR (validation) Number metabolites identified 923 quantified (MS techniques). 2/44 differentiating metabolites from MS validated in NMR.	CHD diagnosis method Yorkshire and Humber Congenital Anomaly Register Database; antenatal, postnatal and GP record linkage. Diagnosis timing Antenatal and postnatal. CHD diagnoses Not specified.	+
Hobbs et al. 2005^30^ Country USA Case control *n* = 224 cases *n* = 90 controls Source Blood (plasma) Sample timing Postnatal (months post delivery): Case median 14.9 months Control median 24.5 months	Case inclusion Arkansas resident during pregnancy and recruitment. Liveborn, stillborn or TOP. CHD (septal, conotruncal, right or left). English or Spanish speaking. Involvement in cohort study. Control inclusion Arkansas resident during pregnancy and recruitment. English or Spanish speaking. No anomaly. Case/control exclusion Aneuploidy/syndrome. Single gene disorder. Pregnant or antiepileptic medicine at recruitment.	Analysis Targeted analysis. HPLC and radioimmunoassay. Number metabolites identified N/A	CHD diagnosis method Echocardiogram, surgery or post mortem. Diagnosis timing Antenatal or postnatal. CHD diagnoses Not specified.	+
Hobbs et al. 2005^31^ Country USA Case control *n* = 331 cases *n* = 125 controls Source Blood (plasma) Sample timing Postnatal (months post‐delivery): Case median 14.9 months Control median 22.8 months	Case inclusion Arkansas resident during pregnancy and recruitment. Liveborn, stillborn or TOP. CHD (septal, conotruncal, right or left). English or Spanish speaking. Involvement in cohort study. Control inclusion Arkansas resident during pregnancy and recruitment. English or Spanish speaking. No anomaly. Case/control exclusion Aneuploidy/syndrome. Single gene disorder. Pregnant or antifolate medicine at recruitment.	Analysis Targeted analysis. HPLC. Number metabolites identified N/A	CHD diagnosis method Echocardiogram, surgery or post mortem. Diagnosis timing Antenatal or postnatal. CHD diagnoses (n) Not specified.	+
Hsu et al.^32^ Country USA Case control *n* = 38 cases *n* = 18 controls Source Blood (plasma) Sample timing Postnatal: Timing not specified	Case inclusion CHD (ToF or HLHS). Involvement in cohort study. Control inclusion No CHD. Age/ethnicyity matched to cases. Case/control exclusion Not specified.	Analysis Targeted analysis by LC‐MS/MS (400 metabolites in panel). Number metabolites identified N/A	CHD diagnosis method Not specified. Diagnosis timing Not specified. CHD diagnoses ToF (22) HLHS (16) 100% EUROCAT Severe CHD.	+

*Note*: Assessment of subject selection, exposure assessment, confounding and statistical analysis classifies studies overall as high quality (++), acceptable (+) or unacceptable (−).

Abbreviations: AVPM, anomalous pulmonary venous malformation; AS, aortic stenosis; ASD, atrial septal defect; AV hypoplasia, atrioventricular hypoplasia; AVSD, atrioventricular septal defect; CHD, congenital heart disease; CMV, cytomegalovirus; DI/LC‐MS, combined direct injection and liquid chromatography and tandem mass spectrometry; DORV, double outlet right ventricle; GC‐MS, gas chromatography mass spectrometry; HLHS, hypoplastic left heart syndrome; HLRS, hypoplastic right heart syndrome; HPLC, high performance liquid chromatography; LC‐MS‐MS, liquid chromatography tandem mass spectrometry; MS, mitral stenosis; NMR, nuclear magnetic resonance; PA, pulmonary atresia; PS, pulmonary stenosis; RAA, right aortic arch; TOP, termination of pregnancy; TA, tricuspid atresia; TGA, transposition of the great arteries; ToF, tetralogy of Fallot; TR, tricuspid regurgitation; VSD, ventricular septal defect.

a Methodology checklist classification derived utilising the Scottish Intercollegiate Guidelines Network (SIGN) case control study risk of bias tool by 2 authors (SM and SR).

### 3.3 Metabolic analysis in pregnancy

Maternal metabolomic analyses was performed in five studies^26^, ^27^, ^28^, ^29^ during the index pregnancy. Methods of metabolomic analysis included gas, liquid and direct injection chromatography coupled with mass spectrometry or NMR spectroscopy. Maternal serum was analysed in two studies.^26^, ^28^ Bahado‐Singh et al. focussed on analysis within the first trimester, limited to isolated CHD in fetuses. All included cases were defined as severe CHD by EUROCAT (European Registry of Congenital Anomalies and Twins). However, the sample size was limited (*n* = 27 cases). Troisi et al. performed analysis with median sampling time in the second trimester. This included pregnancies proceeding to termination in isolated CHD. Seventy cases were included, with 82.9% EUROCAT severe CHD. Taylor et al. performed analysis on maternal plasma in pregnancy with mean sample timing in the second trimester.^33^ Untargeted mass spectrometry‐based metabolomics analysis was performed with NMR validation of metabolites distinguishing cases and controls where possible. However, CHD diagnosis characteristics were not defined.^33^ Two studies^27^, ^29^ performed analysis on maternal urine samples in pregnancy. Friedman et al. analysed samples at a median time point in the second trimester on fetuses with isolated CHD. Case sample size was 36 cases and less than a third of cases were EUROCAT severe diagnoses. Xie et al. performed analysis at a median time point in the third trimester also including fetuses with associated anomalies. 52.9% of the 70 included cases were EUROCAT severe CHD.

**FIGURE 2:**
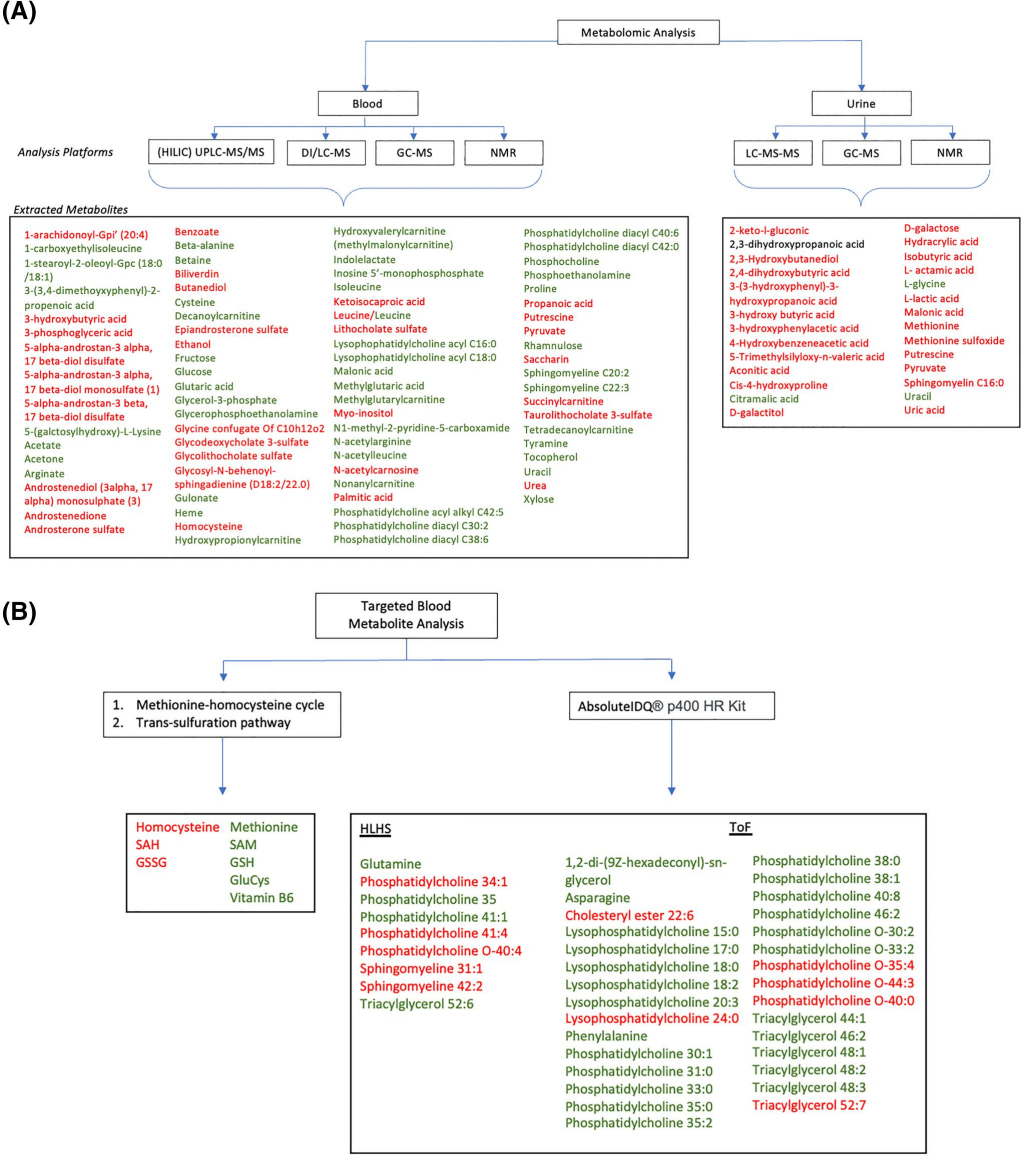
(A): Metabolomic analyses from maternal biosamples taken during pregnancy, synthesised from 5 studies.^26^, ^27^, ^28^, ^29^, ^33^ Metabolites selected through multivariate regression (metabolites significant by *p* < 0.05) or partial least squares discriminant analysis (PLS‐DA) and synthesized from variable importance in projection (VIP) plots (metabolites significant by univariate analysis (*p* < 0.05) included) or selected through volcano plot analysis (*p* < 0.05 and fold change >2 or <−2). Red – metabolite increased in cases versus controls; Green – metabolite decreased in cases versus controls; Black – direction of change unknown. Unnamed (*n* = 7) molecules not included. DI/LC‐MS – combined direct injection and liquid chromatography and tandem mass spectrometry; GC‐MS – gas chromatography mass spectrometry; (HILIC) UPLC‐MS/MS – (hydrophilic interaction liquid chromatography) ultra‐high‐performance liquid chromatography tandem mass spectrometry; NMR – nuclear magnetic resonance; LC‐MS‐MS – liquid chromatography tandem mass spectrometry. (B): Targeted metabolite analysis from maternal blood samples taken out with pregnancy, synthesised from 3 studies.^30^, ^31^, ^32^ Concentration of individual metabolites presented significantly differ (*p* < 0.05) between mothers of CHD cases and controls in confounder adjusted analyses. Red – metabolite increased in cases versus controls; Green – metabolite decreased in cases versus controls. Glucys – Glutamylcysteine; GSH – reduced glutathione; GSSG – oxidized glutathione; HLHS – Hypoplastic left heart syndrome; SAH – S‐adenosylhomocysteine; S‐adenosylmethionine; ToF – tetralogy of Fallot.

Analysis of identified metabolites which significantly differed between case and control groups were assessed by multivariable regression or partial least squares discriminant analysis and synthesised from variable importance in projection plots or selected through volcano plot analysis. Table S1 summarises individual metabolites distinguishing cases and controls with direction of change and method of analysis for each included study. Figure 2A collates metabolites distinguishing case and control groups across included studies. A total of 110 unique metabolites were identified spanning multiple metabolic pathways (excluding 7 unnamed molecules). Differences in lipid metabolism are strongly represented including acylcarnitines, phospholipids, fatty acids, lysophospholipids and sphingolipids, predominantly reduced in cases compared to controls. Metabolites from amino acid, glucose and aerobic metabolism also significantly differ.

Whilst metabolites from similar and related metabolic pathways were identified in multiple included studies, leucine was the only metabolite to significantly differ between cases and controls in two independent studies, with contradictory findings.^28^, ^33^

### 3.4 Mendelian Randomisation

One study utilised three European population cohort studies (*n* = 33,662, 319 CHD cases) to enable Mendelian Randomisation. This analysis allowed further validation of potential causal effects of 27 metabolites differentiating cases and controls in pregnancy (*n* = 44).^33^ From this, 11 metabolites were identified with replication and potential evidence of causality, representing amino acid, steroid lipid and succinylcarnitine metabolic pathways.

### 3.5 Risk prediction models

Risk prediction models were developed in three studies^26^, ^27^, ^28^ in pregnancy utilising metabolites with or without ultrasound markers. Table 2 summarises methods of model development and model performance. Combined screening models for prediction of CHD incorporating metabolite and ultrasound markers outperformed those assessing metabolites alone. The area under the curve was above 0.81 across all models, with a first trimester model combining metabolites (hydroxypropionylcarnitine, glutaconylcarnitine, hydroxytetra‐decadienylcarnitine) with NT having the best performance (AUC 0.992 (95% CI 0.973–1.0), sensitivity 92.9%, specificity 93.2%).^26^

**TABLE 2:** Summary of risk prediction models built utilising multiple metabolites +/− ultrasound markers in pregnancy

Reference	Method of model development	Variables included	Model performance (AUC, 95% CI)
Bahado‐Singh et al. 2014^26^	Logistic regression	Metabolites alone: ‐ Hydroxypropionylcarnitine ‐ Glutaconylcarnitine ‐ Hydroxytetradecadienylcarnitine	0.981 (0.942–0.999) Sensitivity 92.9% Specificity 93.2%
		Metabolites with NT: ‐ Hydroxypropionylcarnitine ‐ Glutaconylcarnitine ‐ Hydroxytetradecadienylcarnitine	0.992 (0.973–1.0) Sensitivity 92.9% Specificity 93.2%
Friedman et al. 2021^27^	Logistic regression	Metabolites alone: ‐ Glutarate ‐ Glucose ‐ Choline ‐ Methionine ‐ Formate ‐ Amniobutyrate	0.815 (0.711–0.919) Sensitivity 80.6% Specificity 78.0%
		Metabolites with 4 chamber view: ‐ Histamine ‐ Choline ‐ Glucose ‐ Formate ‐ Methionine ‐ Carnitine	0.894 (0.814–0.0.973) Sensitivity 83.8% Specificity 87.8%
Troisi et al. 2021^28^	Ensemble machine learning model utilising: ‐ PLS‐DA ‐ Deep learning ‐ Naïve Bayes ‐ Decision tree ‐ Random forest ‐ K‐nearest neighbour ‐ Artificial neural network ‐ Support vector machine ‐ Logistic regression	Metabolites alone: ‐ Individual metabolites not stipulated.	Sensitivity 77.0% +/− 7.0% Specificity 98.0% +/− 1.0%

Abbreviations: AUC, area under curve; NT, nuchal translucency.

### 3.6 Postnatal targeted metabolic analysis

Targeted analysis of metabolites in the B12 and folate dependent homocysteine‐methionine cycle and downstream trans‐sulphuration pathways was performed in two case‐control studies.^30^, ^31^ Both studies utilised participants of the Arkansas Reproductive Health Monitoring System following the index pregnancy. Maternal blood plasma was sampled postnatally, with median sample timings ranging from 14.9 to 24.5 months. CHD diagnosis was made antenatally or postnatally, but individual diagnosis data were not available. Larger sample sizes of 224 and 331 cases were included, respectively. One study performed targeted metabolomic assessment of 400 metabolites (AbsoluteIDQ® p400 HR Kit) in the postnatal period.^32^ Pilot data was available for a small sample of 38 cases (22 tetralogy of Fallot, 16 hypoplastic left heart) and 18 controls. Timings of blood sampling were not available.

Figure 2B synthesises confounder adjusted analysis comparing mean metabolite concentrations between cases and controls. This demonstrates significantly increased mean homocysteine and decreased mean methionine concentrations between cases and controls, in addition to increased downstream metabolites representing sources of oxidative stress. Further, alterations in phospholipid and glutamate metabolic pathways are evidenced, illustrating potential mechanisms of CHD pathogenesis.

### 3.7 Assessment of confounding variables

**FIGURE 3:**
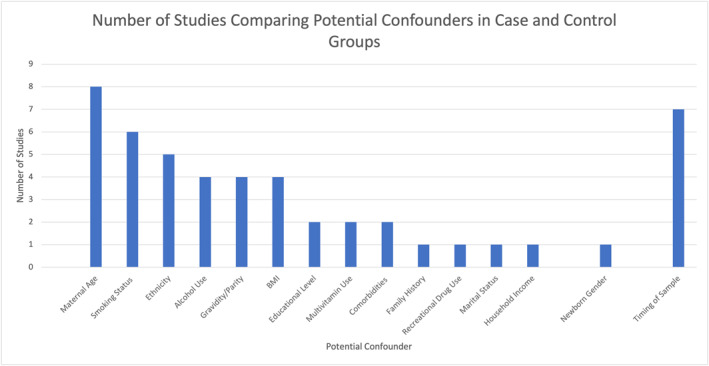
Potential confounding variables compared between case and control groups in included studies, proportion of studies.

Figure 3 summarises potential confounding variables compared between case and control groups across studies. Maternal age was assessed in all studies with timing of biosample analysis in all but one. Maternal smoking status, ethnicity, maternal alcohol consumption, gravidity/parity and maternal BMI were assessed in 50% or more of studies.

## 4 DISCUSSION

### 4.1 Main findings

We present the first systematic literature review in the evolving field of maternal metabolomics in foetal and childhood CHD. Several metabolites distinguishing case and control groups were identified both within pregnancy and the postnatal period. Our findings were limited by study variation with differing timing, origin, methods of sample analysis and heterogenous CHD. However, lipid metabolism, amino acid metabolism and the methionine‐homocysteine cycle and trans‐sulfuration pathway warrant promise for further investigation. Future research should focus on establishing potential underlying maternal risk factor profiling in comparison to putative foetal effects. Several studies built effective predictive models for foetal CHD combining maternal metabolites and ultrasound markers. These offer potential routes to improved screening pathways in the future.

### 4.2 Interpretation

Individual metabolites identified to distinguish cases and controls as summarised in Table S1 and Figure 2 physiologically function and interact in several metabolic pathways.

#### 4.2.1 Lipid metabolism

Lipid metabolites most frequently distinguished maternal metabolic profiles of cases and controls across included studies. Several acylcarnitines and fatty acids, phospholipids including phosphatidylcholine and sphingolipids including sphingomyelin were lower in cases than controls.^26^, ^27^, ^28^, ^29^, ^33^ One study further assessing potential causality through Mendelian randomisation suggested higher levels of the acylcarnitine succinylcarnitine in cases than controls.^33^ As such, understanding the origin of these metabolic differences is essential for future risk profiling, prediction, and mechanistic understanding of foetal CHD. Metabolic profile changes could represent an inherent maternal risk factor for foetal CHD or may point to a process driven by the fetus or placenta.

Acylcarnitines are formed from the binding of carnitine to long chain fatty acids, facilitating transfer of fatty acids across the mitochondrial membrane for lipid metabolism.^34^ Cardiac tissue derives over 95% of its energy from mitochondrial fat metabolism.^35^ Maternal changes in lipid metabolites could reflect alterations in foetal fat metabolism within CHD. This is supported by studies demonstrating higher acylcarnitine concentrations in blood samples of children with VSDs compared to CHD not affecting pulmonary blood flow.^36^ However, such studies include very small sample sizes (*n* = 18). Alternatively, intrinsic alteration in placental metabolism could be reflected in maternal blood. The placenta is known to utilise fatty acids in metabolism, with transplacental transfer of free fatty acids from mother to fetus throughout pregnancy.^37^, ^38^ Altered placental uptake of lipids and changes in lipid deposition are seen in maternal conditions such as obesity.^39^ However, research in this area has not been performed in CHD.

Phosphatidylcholine is the principal phospholipid in human cells. Phospholipids have essential roles including lipoprotein structure, facilitating lipid storage and transfer to prevent lipotoxicity; cell membrane structure for cellular integrity; and mitochondrial membrane structure and modulation of lipid metabolism.^40^ Phosphatidylcholine is synthesised from choline in a S‐adenosylmethionine (SAM)‐dependent reaction, linking synthesis to the methionine‐homocysteine cycle. Choline availability is folate dependent, with requirements increased in pregnancy with placental transfer.^41^ Given the range of important roles discussed, and the links with folate, methionine and homocysteine metabolism, this could relate to inherent maternal risk factors for CHD. Pilot data has suggested similar metabolic changes persist out with pregnancy, potentially supporting this.^32^ Foetal utilisation could further be altered, driving maternal changes; however, data is not available in the context of CHD.

Lysophospholipids are involved in membrane‐derived lipid signalling and mediation.^42^ Sphingolipids such as sphingomyelin have roles in cell cycle, migration, metabolism and protein synthesis.^43^ Studies in zebrafish have shown altered signalling in both metabolite classes affect cardiac development.^43^, ^44^ This is a potential pathway for CHD pathogenesis.

#### 4.2.2 Methionine‐homocysteine cycle and trans‐sulfuration pathway

**FIGURE 4:**
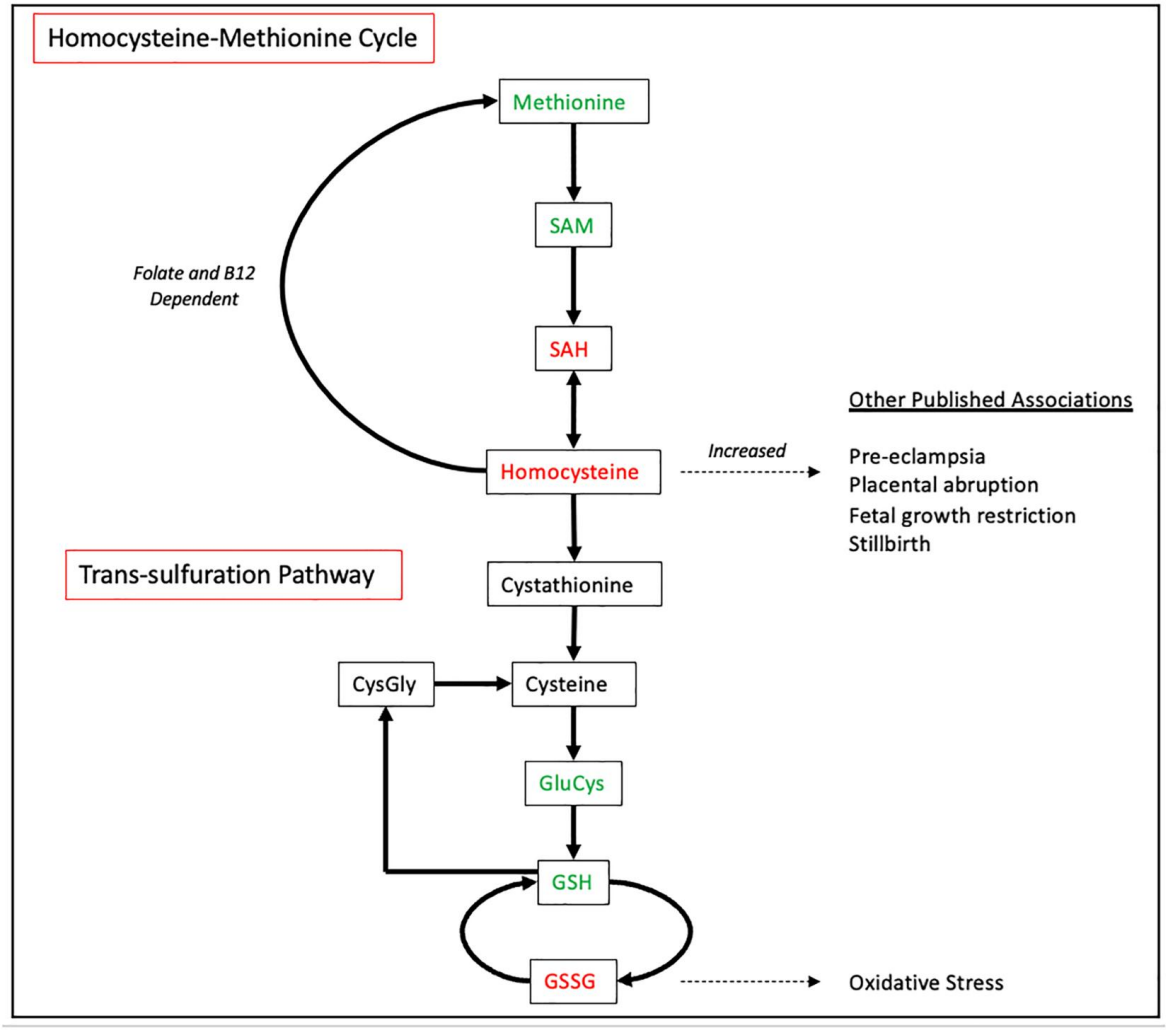
The Homocysteine‐methionine cycle and trans‐sulfuration pathway. Adapted from Hobbs et al. 2005, Tsitsiou et al. 2011.^30^, ^31^, ^47^ Red – metabolite increased in CHD cases versus controls; Green – metabolite decreased in CHD cases versus controls. SAM – S‐adenosylmethionine; SAH – S‐adenosylhomocysteine; GluCys – glutamylcysteine; GSH – reduced glutathione; GSSG – oxidized glutathione; CysGly – cysteinylglycine.

Significant maternal alterations in the methionine‐homocysteine cycle and trans‐sulfuration pathways were identified out with pregnancy, with elevated homocysteine, S‐adenosylhomocysteine (SAH) and oxidised glutathione (GSSG) and lower reduced glutathione (GSH).^30^, ^31^ Figure 4 illustrates the homocysteine‐methionine and transulfuration pathways with published associated outcomes.

Methionine is an essential amino acid, metabolised to homocysteine with SAM and S‐adenosylhomocysteine (SAH) as intermediates. SAH and homocysteine exist in equilibrium, favouring the conversion of homocysteine to SAH.^30^, ^31^ SAH is an inhibitor of cellular methyltransferases, influencing gene expression through methylation during organogenesis.^30^, ^45^ This is a possible mechanism of homocysteine toxicity.^46^ Homocysteine metabolism in the placenta is predominantly folate and B12 dependent conversion to methionine. Raised homocysteine in mothers has been associated with pre‐eclampsia, placental abruption, growth restriction and stillbirth illustrating potential toxicity. Homocysteine can cross the placenta, making foetal effects possible.^47^

Approximately 50% of homocysteine is irreversibly converted to cystathionine to enter the trans‐sulfuration pathway.^31^ Several metabolites within this pathway facilitate oxidative stress through formation of reactive oxidative species.^31^, ^46^ GSH is an antioxidant that under normal conditions exists in a significantly higher concentration than the oxidised form GSSG. A change in the ratio towards GSSG is characterised by excessive oxidative stress.^48^ A shift in this pathway has been seen in animal studies to increase incidence of neural tube defects.^49^ Furthermore, lower GSH has been identified in the rat hyperglycaemic model of induced embryonic malformation.^50^ This process illustrates a potential mechanism of toxicity through the homocysteine trans‐sulfuration pathway.

These findings illustrate potential mechanisms of organotoxicity driven by inherent maternal metabolic alterations through inhibition of cellular methyltransferases and increased oxidative stress. However, mechanistic causative associations have not currently been identified. Furthermore, assessment of maternal profiles was out with the index pregnancy so may not reflect the peri‐conceptual period. However, population cohort studies have shown little variation in homocysteine levels over a period of 1–2 years.^51^

#### 4.2.3 Risk prediction and screening in CHD

Antenatal risk prediction models from included studies utilising combinations of ultrasound and metabolites or metabolites alone obtained sensitivities of 77%–92.9%. The detection rate for major CHD in 2019 utilising existing screening tools was 54.5% in England.^3^ Integrated screening models, including metabolite profiles, demonstrate potential to improve antenatal detection of CHD. However, interpretation of these screening approaches require caution given the small sample sizes included and assessment within samples from which primary analyses had been performed. Furthermore, no single metabolite has consistently distinguished case and control groups in multiple studies, therefore limiting use within clinical practice at present.

#### 4.2.4 A foetal effect?

True alterations in maternal metabolic profiles associated with foetal CHD could represent underlying maternal risk factors, or derive from a foetal effect driven by CHD or the placenta.^21^ Whilst common pathways such as lipid metabolism were identified through included studies, only leucine was replicated in two independent studies with contradictory findings. This makes the foetal effect less likely; however, this cannot be definitively concluded given the small sample sizes and variation of the studies included. At present, established biomarkers utilised in screening for foetal pathologies are often placentally derived, for example, pregnancy associated plasma protein A.^52^ To date, no obvious biomarker has emerged in the included studies within maternal blood or urine for screening of CHD.

A potential method to distinguish maternal metabolic risk profile relative to foetal effect is to assess the maternal metabolome out with the index pregnancy in mothers with offspring affected by CHD. Included studies have assessed the non‐pregnant maternal metabolome utilising targeted platforms.^30^, ^31^, ^32^ Pilot data suggested persistence of altered maternal lipid profiling in the postnatal period, potentially implicating an underlying maternal risk factor profile. However, available evidence is limited. Furthermore, untargeted assessment has not been performed. Longitudinal studies of the metabolome in blood and urine over a period of up to 7 years suggests a high degree of conservation.^53^, ^54^, ^55^, ^56^ Untargeted metabolomic assessment out with pregnancy would provide further clarification on these hypotheses.

### 4.3 Strengths and limitations

This is the first study to systematically review the association between foetal CHD and maternal metabolic alterations. The review has been robustly performed under PRISMA guidelines with clear inclusion criteria. However, it is limited by the variation of studies. Across studies, case sample size was limited, with variation in CHD diagnoses included. CHD was diagnosed across a variety of time points; antenatal, postnatal and at post‐mortem setting. Given the range of studies included with differing timing, origin, methods of sample analysis and heterogenous CHD, quantitative synthesis of data was precluded. Furthermore, inclusion of observational studies introduces inherent risk of bias and confounding. CHD in the included studies varied in severity and classification, with EUROCAT defined severe CHD between 27.8% and 100%. Three studies did not specify diagnosis data, again limiting the generalisability of findings.^30^, ^31^, ^33^

### 4.4 Conclusions

At present, there is not enough available evidence to utilise maternal metabolomic biomarkers as screening tools for CHD in clinical practice. In addition, further research is required to identify maternal metabolic risk factors and establish biomarker identification and validation across different populations. Delineating maternal risk profiles and a potential foetal effect remains a priority in future research, which can be addressed through metabolomic assessment with index pregnancies. This review identifies several metabolic pathways that illustrate potential CHD pathogenic mechanisms. In addition, the development of combined screening tools using metabolites and ultrasound features is an exciting prospect for future research.

## CONFLICT OF INTEREST

The authors declare they have no conflicts.

## Supporting information

Figure S1

Table S1

## Data Availability

The data that support the findings of this study are available from the corresponding author (SM), upon reasonable request.
