# Supplementary material for: Maternal metabolomic profiling and congenital heart disease risk in offspring: A systematic review of observational studies
Source: Prenat Diagn. 2023 Jan 26;43(5):647–60. doi: 10.1002/pd.6301 (PMC10946495; doi:10.1002/pd.6301)
Supplement: Supplementary file 1 — Figure S1 [file PD-43-647-s001.docx]

OVID-Embase


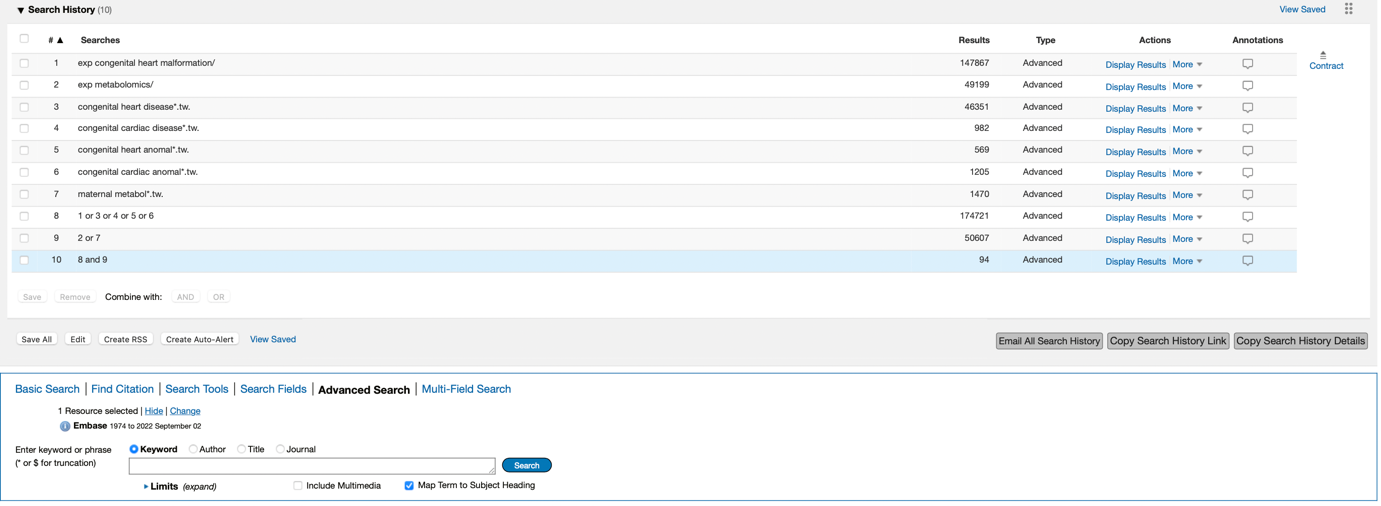


OVID-MEDLINE


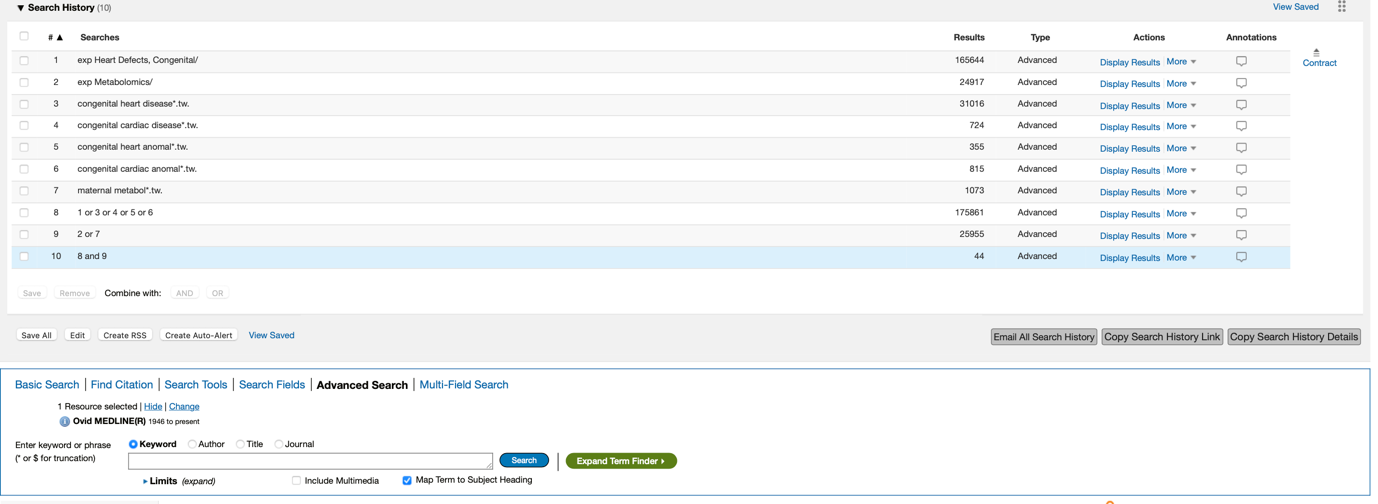


Cochrane Library
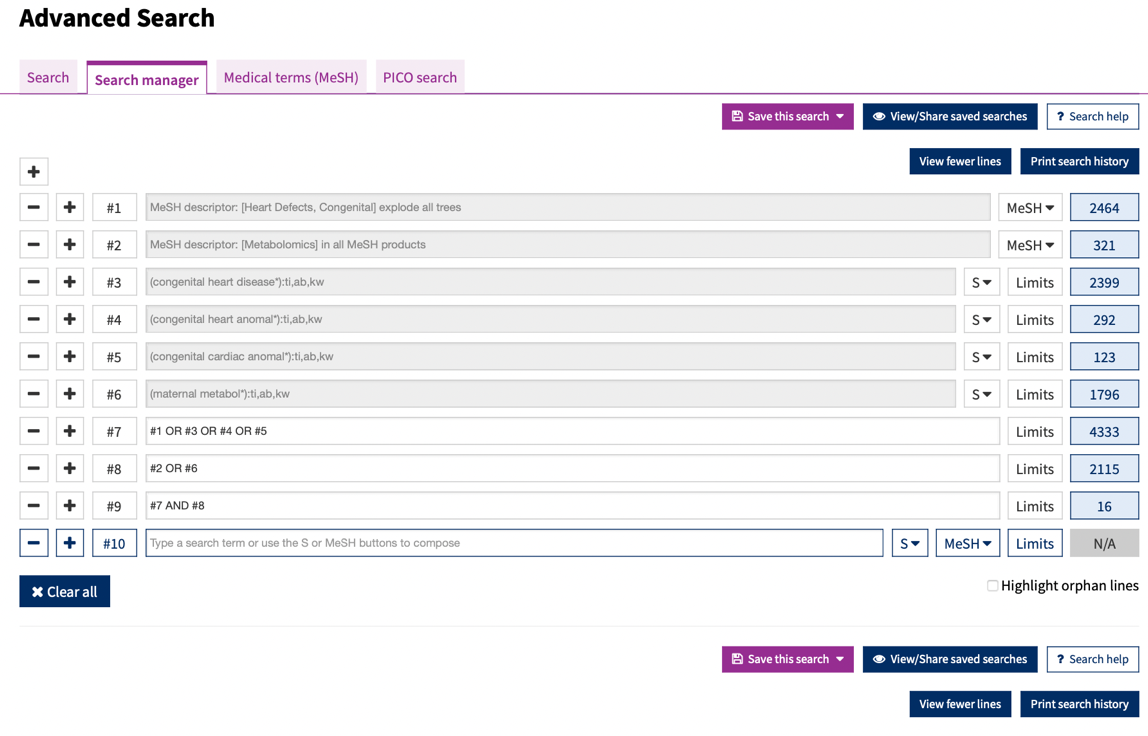


*Figure S1: Search strategy for each database – OVID-Embase, OVID-MEDLINE and Cochrane Library.*
