# Supplementary material for: Maternal metabolomic profiling and congenital heart disease risk in offspring: A systematic review of observational studies
Source: Prenat Diagn. 2023 Jan 26;43(5):647–60. doi: 10.1002/pd.6301 (PMC10946495; doi:10.1002/pd.6301)
Supplement: Supplementary file 2 — Table S1 [file PD-43-647-s002.docx]

**Supplementary Table 1**

*Table S1:* *Individual metabolites distinguishing case and control groups with direction of change and method of analysis for each included study.*

| **Reference** | **Method of analysis identifying metabolites distinguishing cases and controls** | **Metabolite Increased in Cases** | **Metabolite Decreased in Cases `Green** |
| --- | --- | --- | --- |
| Bahado-Singh et al. 2014  (26) | Variable Importance in Projection Plot (individual metabolites also significant on univariate analysis) | Ethanol  Pyruvate | Acetate  Acetone  Decanoylcarnitine (c10)  Hydroxypropionylcarnitine (C3-OH)  Hydroxyvalerylcarnitine (methylmalonylcarnitine)  Lysophophatidylcholine acyl C16:0  Lysophophatidylcholine acyl C18:0  Methylglutarylcarnitine (C5-M-DC)  Nonanylcarnitine (C9)  Phosphatidylcholine acyl alkyl C42:5  Phosphatidylcholine diacyl C30:2  Phosphatidylcholine diacyl C38:6  Phosphatidylcholine diacyl C40:6  Phosphatidylcholine diacyl C42:0  Sphingomyeline C20:2  Sphingomyeline C22:3  Tetradecanoylcarnitine (C14:1) |
| Friedman et al. 2021  (27) | Variable Importance in Projection Plot (individual metabolites also significant on univariate analysis) | Methionine  Isobutyric acid  Putrescine  L-lactic acid  cis-4-hydroxyproline  Methionine sulfoxide  Sphingomyelin C16:0 |  |
| Troisi et al. 2020  (28) | Variable Importance in Projection Plot and Volcano Plot | 3-hydroxybutyric acid  3-phosphoglyceric acid  Androstenedione  Butanediol  Homocysteine  Ketoisocaproic acid  Leucine  Palmitic acid  Propanoic acid  Putrescine  Urea | 3-(3,4-dimethoxyphenyl)-2-propenoic acid  Beta-alanine  Cysteine  Fructose  Glucose  Glutaric acid  Malonic acid  Methylglutaric acid  Proline  Rhamnulose  Tocopherol  Tyramine  Uracil  Xylose |
| Xie et al. 2019  (29) | Variable Importance in Projection Plot (individual metabolites also significant on univariate analysis) | 2-keto-l-gluconic  2,3-dihydroxypropanoic acid  2,3-Hydroxybutanediol  2,4-dihydroxybutyric acid  3-(3-hydroxyphenyl)-3-hydroxypropanoic acid  3-hydroxy butyric acid  3-hydroxyphenylacetic acid  4-Hydroxybenzeneacetic acid  5-Trimethylsilyloxy-n-valeric acid  Aconitic acid  D-galactitol  D-galactose  Hydracrylic acid  L- actamic acid  Malonic acid  Pyruvate  Uric acid | Citramalic acid  L-glycine  Uracil |
| Taylor et al. 2022  (33) | Confounder adjusted multivariable regression | 1-arachidonoyl-Gpi' (20:4)  17beta- diol disulphate  17beta- diol disulphate  17beta- diol monosulphate (1)  5alpha-androstan-3alpha  5alpha-androstan-3alpha  5alpha-androstan-3beta  Androstenediol (3 alpha, 17 alpha) monosulphate (3)  Androsterone sulphate  Benzoate  Biliverdin  Epiandrosterone sulphate  Glycine conjugate Of C10h12O2  Glycodeoxycholate 3-sulphate  Glycolithocholate sulphate  Glycosyl-N-behenoyl-sphingadienine (D18:2/22:0)  Lithocholate sulphate (1)  Myo-inisitol  N-acetylcarnosine  Saccharin  Succinylcarnitine  Taurolithocholate 3-sulphate  X-18921  X-22520  X-24544  X24947 | 1-carboxyethylisoleucine  1-stearoyl-2-oleoyl-Gpc (18:0/18:1)  5-(galactosylhydroxy)-L-lysine  Argininate  Betaine  Glycerol-3-phosphate  Glycerophosphoethanolamine  Gulonate  Heme  Indolelactate  Insosine 5'-monophosphate (Imp)  Isoleucine  Leucine  N-acetylleucine  N-acteylarginine  N1-methyl-2-pyridone-5-carboxamide  Phosphocholine  Phosphoethanolamine  X-11787  X-19438  X-24295 |
| Hobbs et al. 2005  (30) | Linear regression | Homocysteine  S-adenosylhomocysteine | Methionine  S-adenosylmethionine |
| Hobbs et al. 2005  (31) | Linear regression | Homocystine  Oxidised glutathione (GSSG) | Glutamylcysteine  Reduced glutathione (GSH)  Vitamin B6 |
| Hsu et al. 2022  (32) | Analysis of covariance (ANCOVA) | Hypoplastic Left Heart Syndrome:  Phosphatidylcholine 34:1  Phosphatidylcholine 41:4  Phosphatidylcholine O-40:4  Sphingomyeline 31:1  Sphingomyeline 42:2  Tetralogy of Fallot:  Cholesteryl ester 22:6  Lysophosphatidylcholine 24:0  Phosphatidylcholine O-35:4  Phosphatidylcholine O-44:3  Phosphatidylcholine O-40:0  Triacylglycerol 52:7 | Hypoplastic Left Heart Syndrome:  Glutamine  Phosphatidylcholine 35  Phosphatidylcholine 41:1  Triacylglycerol 52:6  Tetralogy of Fallot:  1,2-di-(9Z-hexadeconyl)-sn-glycerol  Asparagine  Lysophosphatidylcholine 15:0  Lysophosphatidylcholine 17:0  Lysophosphatidylcholine 18:0  Lysophosphatidylcholine 18:2  Lysophosphatidylcholine 20:3  Phenylalanine  Phosphatidylcholine 30:1  Phosphatidylcholine 31:0  Phosphatidylcholine 33:0  Phosphatidylcholine 35:0  Phosphatidylcholine 35:2  Phosphatidylcholine 38:0  Phosphatidylcholine 38:1  Phosphatidylcholine 40:8  Phosphatidylcholine 46:2  Phosphatidylcholine O-30:2  Phosphatidylcholine O-33:2  Triacylglycerol 44:1  Triacylglycerol 46:2  Triacylglycerol 48:1  Triacylglycerol 48:2  Triacylglycerol 48:3 |
